# Evaluating the Utility of Wearable Sensors for the Early Diagnosis of Parkinson Disease: Systematic Review

**DOI:** 10.2196/69422

**Published:** 2025-07-21

**Authors:** Hai Li, Massimiliano Zecca, Jiajun Huang

**Affiliations:** 1 College of Sport Neijiang Normal University Neijiang China; 2 School of Mechanical, Electrical and Manufacturing Engineering Loughborough University Loughborough United Kingdom; 3 Department of Neurology The Second People's Hospital of Neijiang Neijiang China

**Keywords:** wearable sensor, Parkinson disease, early diagnosis, digital biomarkers, motor symptoms

## Abstract

**Background:**

Early diagnosis is crucial for ensuring that patients with Parkinson disease (PD) receive timely treatment, which can improve their quality of life and prolong lifespan. Wearable sensors have emerged as promising tools for early PD diagnosis, offering noninvasive, continuous symptom monitoring.

**Objective:**

This review aimed to evaluate how wearable sensors have been applied in early diagnosis of PD over the past decade, focusing on sensor types, methods, findings, and limitations.

**Methods:**

The systematic review was conducted following the PRISMA (Preferred Reporting Items for Systematic Reviews and Meta-Analyses) guidelines. Studies were sourced from PubMed, IEEE Xplore, Scopus, and Web of Science and screened based on predefined criteria. The inclusion criteria were as follows: (1) the study was observational or experimental, (2) wearable sensors were applied for the early diagnosis of PD, (3) participants were diagnosed with early-stage or prodromal PD, (4) the study included at least 10 participants with PD, and (5) the article was published between 2013 and 2023. Studies were excluded if they focused solely on treatment, rehabilitation, symptom monitoring, or nonwearable devices; lacked diagnostic clarity; were not published in English; or were not primary research articles. All the selected studies were assessed for quality using the Quality Assessment of Diagnostic Accuracy Studies-2, the quality assessment tool recommended by the Cochrane Collaboration.

**Results:**

Overall, 1888 records were retrieved from the selected databases, with 1044 records remaining after duplicate removal. Following the screening of titles and abstracts, 949 ineligible records were excluded, leaving 95 articles for eligibility. Eventually, of the 1044 studies, 12 (1.12%) met the inclusion criteria, validating the feasibility of wearable sensors in the early diagnosis of PD. Most (10/12, 83%) were cross-sectional studies, with 1 longitudinal and 1 mixed-design study. Of the 12 studies, 4 (33%) focused on identification diagnosis, 2 (17%) addressed the staged diagnosis of PD, and 1 (8%) focused on the identification of specific symptoms. Of the 12 studies, 5 (42%) assessed the overall feasibility and performance of wearable sensors in early PD detection without targeting specific classification purposes. The main wearable sensors used were inertial measurement units (8/12, 67%) and accelerometers (4/12, 33%), which primarily captured motion-related data. While initial findings suggest that wearable sensors are feasible for early PD diagnosis, the evidence is still limited by small sample sizes and short study durations.

**Conclusions:**

Wearable sensors show promise in supporting the early diagnosis of PD, particularly for motor symptoms monitoring. However, several limitations remain in validating and applying wearable sensors in clinical contexts, including cross-sectional designs and limited diagnostic standardization. More diverse studies are needed to further validate these findings and address existing shortcomings to better advance the use of wearable sensors in the early diagnosis of PD.

**Trial Registration:**

PROSPERO CRD42024544198; https://www.crd.york.ac.uk/PROSPERO/view/CRD42024544198

## Introduction

### Background

Parkinson disease (PD), an age-related brain disorder characterized by the progressive loss of dopamine-producing nerve cells in the substantia nigra, is considered one of the most common neurodegenerative diseases and can cause motor, cognitive, sleep, and other health issues that can severely impair the quality of life of patients with PD [1-3]. While the exact causes of PD are not fully understood, age is widely recognized as a significant factor associated with the condition. The risk of developing PD increases substantially with advancing age, particularly after reaching the age of 60 years [1,3]. As the world accelerates into an era of population aging, the incidence of PD is on the rise year by year. The World Health Organization reported in 2023 that the global incidence of PD has doubled over the past 25 years [2]. The report estimates that >8.5 million people were living with PD globally in 2019, with approximately 5.8 million experiencing the impact measured by disability-adjusted life years. In addition, there has been a significant increase in deaths attributed to PD, with 329,000 deaths in 2019—an increase of >100% since 2000. A 2022 study in the United States estimated that approximately 1 million people were living with PD in the country that year, with around 90,000 new cases expected to be diagnosed annually [4]. Meanwhile, researchers in China have predicted that the number of people with PD in the country will reach 5 million by 2030, representing about half of the global patient population [5]. The number of PD cases will rise significantly as the world rapidly enters an aging phase.

PD significantly affects the quality of life of patients and reduces their life expectancy [3]. In a meta-analysis involving 2707 patients with PD and 150,661 healthy controls, Zhao et al [6] found that the quality of life among participants with PD was lower than that of healthy individuals across most domains, particularly in physical functioning and mental health. Despite advances in treatment, PD still has a significant impact on life expectancy. A 2018 study showed that the average life expectancy from onset to death for people with PD, based on an average age of onset of 60 years, was approximately 14.6 years, compared with 23.3 years for the general population of the same age [7]. In addition, the costs associated with treatment and rehabilitation are considerable, placing immense financial pressure on patients’ families and society [8]. For example, a study published in 2020 by Gomez-Inhiesto et al [9] showed that the total cost of 5 years of treatment for a patient with PD in Spain was as follows: €53,217 (US $62,365) for deep brain stimulation, €208,163 (US $243,979) for continuous duodenal levodopa or carbidopa infusion, and €170,591 (US $199,942) for continuous subcutaneous apomorphine infusion. In addition, a study conducted in the United States showed that the total economic burden of PD reached US $51.9 billion in 2017, with projections estimating that this figure will exceed US $79 billion by 2037 [10].

Although researchers have still not found any treatments to stop or reverse the progression of PD [11,12], studies have demonstrated that early diagnosis and preventive treatment of PD can extend the life expectancy of patients and provide benefits in terms of symptom control, disease progression, and overall quality of life [13,14]. For example, early treatment with drugs such as dopamine agonists and monoamine oxidase B inhibitors significantly reduces motor symptoms and delays the need for levodopa in patients with PD, potentially avoiding the long-term complications associated with levodopa use [14,15]. Besides, early treatment can also effectively control nonmotor symptoms, such as depression and cognitive decline, that typically occur in the early stages of PD, thereby maintaining or improving patients' quality of life [16]. However, despite the numerous benefits of early treatment for PD, most patients in clinical practice are still diagnosed and commence treatment only after significant symptoms have manifested, typically in the mid to late stages of the disease [17,18]. In the mid to late stages, a significant portion of specific neurons generally have degenerated, resulting in a loss of neural plasticity [13]. Consequently, clinical interventions at this juncture may be less efficacious, as the diminished neural plasticity limits the capacity for neural repair or compensation [19]. Therefore, developing and studying techniques and methods for early diagnosis of PD are essential for effective treatment.

Researchers believe that in the early stages of PD, before typical clinical motor symptoms appear, some presymptoms caused by PD will appear, such as loss or decrease of sense of smell, sleep disturbances, constipation, tremor, and slowing of movement [17,20]. Currently, the clinical diagnosis of PD relies on clinician assessments based on the Movement Disorder Society Clinical Diagnostic Criteria for Parkinson’s Disease (MDS-PD criteria), often supplemented by imaging tools such as DaTSCAN [21,22]. Although effective, these diagnostic approaches are resource intensive and typically applied only after patients present with motor symptoms, limiting their role in early detection [22]. Therefore, in recent years, efforts have been made to enhance the early diagnosis of PD by monitoring prodromal symptoms using new technologies, such as biomarkers, imaging techniques, sensor technologies, and artificial intelligence (AI), with some promising research results being achieved. For example, a study by Pavelka et al [23] shows the potential use of peripheral blood microRNA profiles as diagnostic indicators for distinguishing between PD and progressive supranuclear palsy. Prashanth et al [24] developed a prediction or prognostic model using the support vector machine (SVM) to classify striatal binding ratio values calculated from ^123^I-Ioflupane (DaTSCAN) single photon emission computed tomography scans, achieving 96% accuracy in screening the early stage of PD from healthy individuals in the study. Finberg et al [25] developed an electronic sensor capable of distinguishing the individuals with early-stage PD from healthy individuals by detecting volatile molecules in exhaled breath.

Wearable sensors are particularly interesting among the various technologies due to their noninvasive, continuous, and real-time monitoring capabilities. Wearable sensors extend monitoring beyond the clinical setting into real-world environments, enabling the collection of more comprehensive data [26,27]. As a result, wearable sensors can capture transient symptoms that are often difficult to detect during regular clinical examinations, which has significant implications for the diagnosis and monitoring of PD [27]. In addition to diagnostic utility, wearable sensors offer substantial cost-effectiveness by reducing the frequency of hospital visits and enabling remote, long-term monitoring [28]. These benefits are particularly valuable in aging populations and in low-resource settings where access to specialized neurological care is limited [29]. Recent studies have demonstrated the effectiveness of wearable sensors in diagnosing and monitoring PD symptoms in later stages. For instance, inertial measurement units (IMUs), which consist of accelerometers, gyroscopes, and sometimes magnetometers [30,31], embedded in wristbands or smartwatches can effectively track the frequency and amplitude of tremors in patients with PD, providing a detailed understanding of the severity of their motor symptoms [32]. In addition, the study has focused on monitoring and detecting freezing of gait, a common and severe symptom in patients with PD, by placing sensors on the lower limbs [33]. Moreover, researchers have also explored the use of IMUs to monitor motor symptoms and assist in the differential diagnosis of PD and other disorders with similar clinical presentations [34,35]. These studies demonstrate the use of wearable sensors such as smartwatches with embedded IMUs and sensor-equipped insoles for monitoring symptoms and supporting the diagnosis of PD.

In the realm of early diagnosis of PD, researchers have tried to experiment with wearable sensors, such as those embedded in smartphones, to monitor individuals’ daily behaviors to identify early symptoms of the disease [36]. However, research in this field remains in the exploratory stage. A review [37] of the application of wearable sensors in PD from a few years ago highlighted that studies on the application of wearable sensors for early diagnosis of PD were quite limited.

### Objectives

Several reviews in recent years have examined wearable sensors or platforms in the context of PD, but most have focused on technologies for symptom monitoring and treatment or have only briefly addressed early diagnosis [37]. However, a comprehensive systematic review specifically focused on early diagnosis is still lacking. In addition, there were very few studies involving the application of wearable sensors for the early diagnosis of PD before 2013 [37]. There remains a gap in systematically reviewing the use of wearable sensors specifically for the early diagnosis of PD. Given the advances in sensor and AI technologies over the past decade, this systematic review aims to fill this gap by evaluating recent research (2013-2023) on wearable sensors in the early diagnosis of PD, addressing the following key research questions (RQs):

- RQ 1: What types of wearable sensors are being used for the early diagnosis of PD?
- RQ 2: What methods and technologies are used in these studies, including wearing styles, types of data acquired, and methods of data analysis and processing?
- RQ 3: What are the accuracy, reliability, utility, and limitations of wearable sensors in the early diagnosis of PD?

### Key Terms in This Review

In this review, wearable sensors are defined as platforms that collect data continuously in real time without significantly affecting the wearer’s activities [38-40]. Therefore, our review specifies that wearable sensors should be easy to wear and operate, capable of transmitting data wirelessly, or equipped with self-contained memory to store data. It is also worth noting that smartphones have been widely used in medical research as atypical wearable platforms [41,42]. In this context, sensors integrated into smartphones that can collect real-time data and respond to human body–related input were classified as wearable sensors.

As for PD, the included studies were required to specifically involve patients with early PD or pre-PD. To identify early stages of PD, we combined the diagnostic criteria in the United Kingdom [43], China [44], and Europe [45] and considered stages 0 to 2.5 on the Hoehn and Yahr (H&Y) scale. Considering that some studies may not provide the H&Y scale scores of patients and may use Movement Disorder Society-Unified Parkinson’s Disease Rating Scale (MDS-UPDRS) scores instead, this study refers to the study by Skorvanek et al [46] to convert MDS-UPDRS scores into H&Y scale scores to determine the stage of PD symptoms.

## Methods

### Search Strategy

This systematic review followed the PRISMA (Preferred Reporting Items for Systematic Reviews and Meta-Analyses) guidelines (Multimedia Appendix 1 for the checklist) and was registered with the PROSPERO international database (CRD42024544198). Electronic databases relevant to the field—IEEE Xplore, PubMed, Scopus, and Web of Science—were searched for English-language articles published (including prepublication) between January 1, 2013, and December 31, 2023.

The search strategy included terms related to PD, wearable sensors, and early diagnostic methods combined with Boolean operators, which were searched as follows: (“Parkinson” OR “pre-Parkinson”) AND (“wearable” OR “inertial” OR “accelerometer” OR “acceleration” OR “gyroscope” OR “EMG” OR “ EEG” OR “ECG” OR “GSR” OR “pressure” OR “clothes” OR “smartphone” OR “smartwatch” OR “glove”) AND (“early diagnosis” OR “early detection” OR “early stage” OR “timely diagnosis” OR “early identification” OR “prediagnostic” OR “prompt diagnosis” OR “initial detection” OR “anticipatory diagnosis” OR “prodromal” OR “presymptomatic” OR “preclinical” OR “incipient” OR “pre-manifest” OR “subclinical” OR “latent”). Instead of using “IMU,” we deliberately selected its core component terms—“inertial,” “accelerometer,” “acceleration,” and “gyroscope”—to construct a more inclusive search strategy. This approach ensured that studies involving IMU-related technologies, even those not explicitly using IMU, were captured under broader terminology. The search strategy is provided in Multimedia Appendix 2. In addition to searching the selected databases, the reference lists of the included papers were manually checked to determine if any relevant studies were missed.

### Selection

The process was conducted in 2 distinct stages: title and abstract screening and full-text review. Both stages were performed independently by 2 researchers (HL and JH), and any discrepancies were resolved through discussion with a third researcher (MZ). The screening criteria for the studies are listed in Textbox 1.

**Eligibility criteria.**

**Inclusion criteria**

- Were observational (cross-sectional, case-control, or cohort) or experimental studies (randomized controlled trials or laboratory-based studies)
- Applied wearable sensors for the early diagnosis of Parkinson disease (PD)
- Included participants diagnosed with early-stage PD or prodromal PD
- Involved at least 10 participants with PD
- Were published between 2013 and 2023
- Were written in English with full text available

**Exclusion criteria**

- Focused solely on treatment, rehabilitation, or symptom monitoring
- Used nonwearable or clinically tethered devices
- Lacked clearly defined PD diagnostic criteria or stage classification
- Had a sample size of <10 participants with PD
- Were review articles, editorials, conference abstracts, or non-English publications

Studies with <10 participants diagnosed with PD were excluded from this review based on considerations of clinical relevance and statistical interpretability. Given that this review focuses on the potential of wearable sensors for early diagnosis of PD, studies with very small samples may not be able to provide clinically meaningful findings [47,48]. Therefore, a minimum participant threshold was used in this review to increase the reliability and representativeness of the included studies.

### Data Extraction

Data extraction was performed using a standardized form and included the following information: details on wearable sensors and platforms, population characteristics, assessment tasks, methods of data analysis, and the performance of early PD diagnosis. Data extraction was conducted by one researcher (HL) and checked by another (JH), and any disagreements were resolved by consensus with a third researcher (MZ).

### Data Synthesis

This study adopts a narrative synthesis approach and does not include a meta-analysis. Given the anticipated heterogeneity in study designs, sensor modalities, analytical methods, and outcome measures, the included studies were grouped and descriptively compared according to predefined analytical dimensions: (1) types and placement of wearable sensors, (2) platforms used to collect and process sensor data, (3) population characteristics (eg, sample size, age, and disease stage), (4) assessment tasks, (5) data analysis approaches (eg, feature extraction methods and machine learning models), and (6) diagnostic performance metrics (eg, accuracy, sensitivity, and specificity). Data were extracted using structured summary tables and synthesized qualitatively to identify methodological trends, strengths, and limitations. Statistical pooling or effect size integration was not performed due to the diversity of outcome measures across studies. Data synthesis was conducted by one researcher (HL) and checked by another (JH), and any disagreements were resolved by consensus with a third researcher (MZ).

### Quality Assessment

#### Overview

In this review, the quality of the included studies was assessed using the Cochrane-recommended Quality Assessment of Diagnostic Accuracy Studies-2 (QUADAS-2) [49]. QUADAS-2 evaluates the risk of bias and applicability concerns in 4 key domains: patient selection, index test, reference standard, and flow and timing. Quality assessment was conducted by one researcher (HL) and checked by another (JH), and any disagreements were resolved by consensus with a third researcher (MZ).

#### Patient Selection

In this review, patient selection focused on clearly defining the criteria for inclusion and exclusion, including factors such as age, clinical presentation, and stage of PD. In addition, the method of recruiting patients (eg, consecutive or randomized recruitment) was considered to ensure that the recruited patients were representative of the target population, thereby reducing the risk of bias.

#### Index Test

The assessment of the index test focused on whether the study included detailed information about the metrics acquired by the wearable sensor. This information includes sensor type, specifications, measurement parameters, and data analysis methods. Particular attention was given to data analysis methods, ensuring that measures such as data separation or cross-validation were used to reduce bias [50,51]. In addition, the results of the index test needed to be validated in conjunction with clinical references for PD (eg, neurologist’s diagnosis) to improve reliability.

#### Reference Standard

As the application of wearable sensors in the early diagnosis of PD is still in the exploratory research stage and lacks mature clinical diagnostic criteria, the clinical diagnostic criteria for MDS-PD criteria [21] or the clinical diagnosis by neurologists were used as the gold standard reference.

#### Flow and Timing

Detailed information about the patient flow through the various study phases, including information about patient withdrawal and its handling, was considered in the assessment of process and timing. In addition, the time interval between wearable sensor tests and the patient’s clinical diagnosis was examined to ensure that the timing of the studies was rational and consistent.

## Results

### Overview

**Figure 1:**
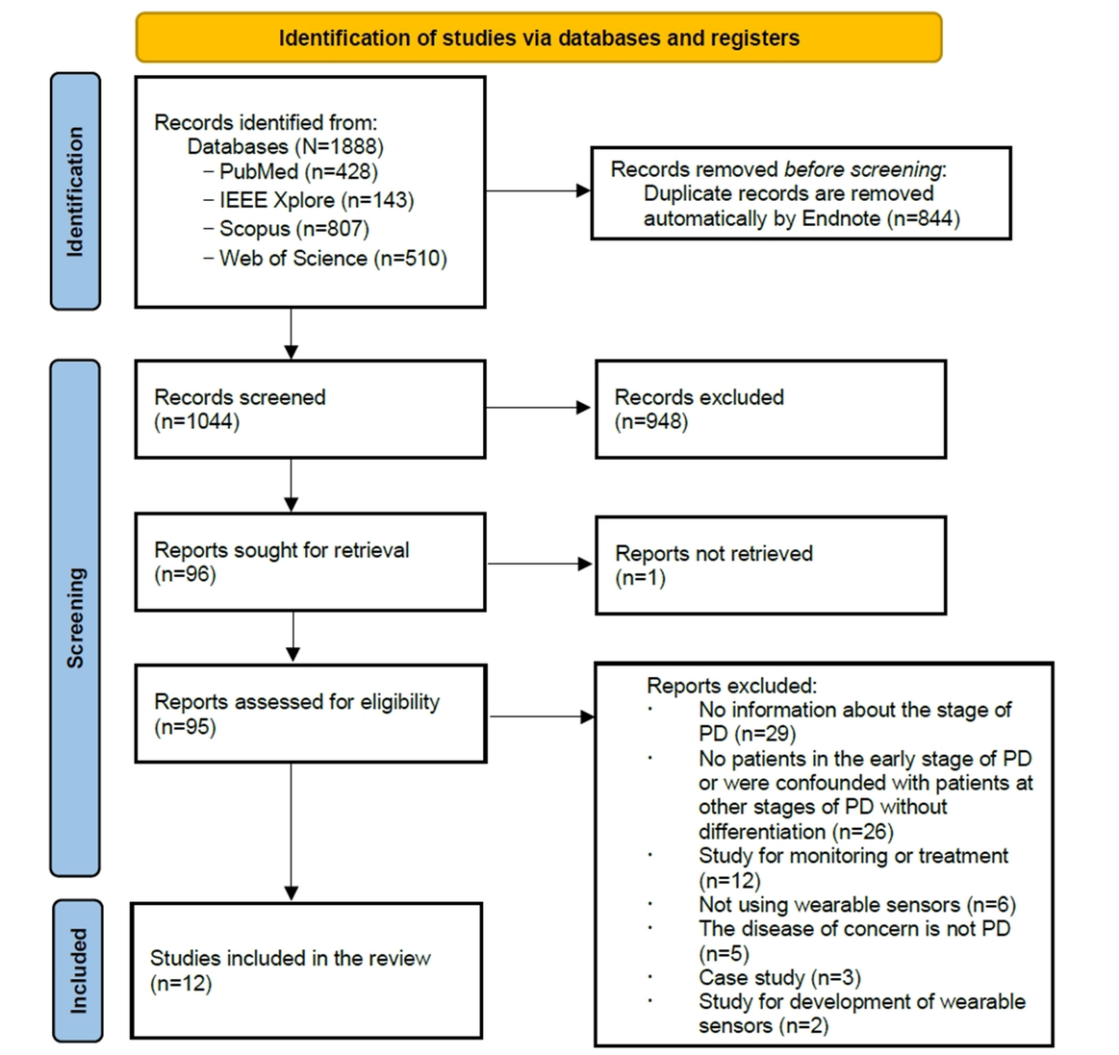
Flowchart of the search and study selection. PD: Parkinson disease.

A total of 1888 records were identified by screening PubMed (n=428), IEEE Xplore (n=143), Scopus (n=807), and Web of Science (n=510). After removing 844 duplicates using EndNote 20 (Clarivate), 1044 records remained for screening. A total of 949 records were excluded by screening titles and abstracts, and 95 (95/1044, 9.1%) reports were assessed for eligibility. Among these reports, 83 reports were excluded for various reasons, including (1) lack of information on the stage of PD, (2) no patients in the early stage of PD or were confounded with patients at other stages of PD without differentiation, (3) study for monitoring or treatment, (4) not using wearable sensors, (5) the disease of concern is not PD, and (6) number of participants diagnosed with PD is <10. Ultimately, of the 1044 studies, 12 (1.15%) met the criteria for inclusion in the review. The information on the screening process is shown in the PRISMA flowchart [52] (Figure 1) and adheres to the PRISMA 2020 guidelines (Multimedia Appendix 1). Extracted information for each of the selected studies is shown in Table 1. Notably, of the 83 reports that were excluded, 55 were omitted due to issues related to the experimental design involving participants. Specifically, 29 reports were excluded due to a lack of information on the staging of PD, while 26 were excluded because they failed to include patients in the early stage of PD or were confounded with patients at other stages of PD without differentiation.

**Table 1:** Summary of data extraction.

Study	Wearable sensors and placement	Platforms	Population characteristics	Assessment tasks	Data analysis	Performance
Arora et al [53]	Sensors: IMUs^a^ (fs^b^=20 Hz), microphone, touch screen sensors, and timer Placement: hands and pelvis	Smartphone	HC^c^ (56 male and 28 female; age: mean 66.3, SD 9.1 years; H&Y^d^ scale score: mean 0, SD 0.2) iRBD^e^ (91 male and 13 female; age: mean 64.5, SD 9.4 years; H&Y scale score: mean 0.1, SD 0.4) PD^f^ (209 male and 125 female; age: mean 66.1, SD 9.0 years; H&Y scale score: mean 1.8, SD 0.5)	7 tasks developed by researchers: voice, balance, gait, finger tapping, reaction time, rest tremor, and postural tremor	Features: features of voice impairment; temporal and spatial features from finger tapping, reaction time, gait, and balance features; and tremor feature Algorithm and model: RF^g^	HC and PD: sensitivity: 84.6% and specificity: 88.3% HC and iRBD: sensitivity: 91.9% and specificity: 90.0% PD and iRBD: sensitivity: 87.5% and specificity: 90.1%
Rovini et al [54]	Sensors: IMUs (fs=100 Hz) Placement: hands, fingers, and feet	SensHand V1 (hands and fingers) and SensFoot V2 (feet)	HC (11 male and 4 female; age: mean 64.9, SD 2.8 years) IH^h^ (10 male and 5 female; age: mean 66.5, SD 2.9 years) PD (7 male and 8 female; age: mean 66.6, SD 7.1; H&Y scale score: mean 1.3, SD 0.5)	10 tasks from MDS-UPDRS III^i^: 4 items for lower limbs (toe tapping, leg agility, rotation, and gait) and 6 items for upper limbs (thumb-forefinger tapping, hand opening-closing, pronation and supination, hand resting tremor, postural tremor, and arms swing during the gait)	Features: spatiotemporal and frequency features Algorithm and model: RF	HC and PD: accuracy: 100% and specificity: 100% HC and PD and IH: accuracy: 91.1% and specificity: 95.6%
Borzí et al [36]	Sensors: IMUs (fs=200 Hz) Placement: lower back	Smartphone	HC (5 male and 2 female; age: mean 27.2, SD 2 years) PD (age: mean 68.6, SD 10.7 years; class 0: 10 male and 6 female; H&: 1-2; class 1: 10 male and 5 female; H&Y scale score: 2; class 2: 9 male and 2 female; H&Y scale score: 3)	Quiet stance for 30 seconds	Features: time and frequency features Algorithm and model: SVM^j^	HC and class 0: accuracy 100%, sensitivity 100%, and specificity 100% Class 0 and class 1: accuracy 82.8%, sensitivity 85.7%, and specificity 80.0%, class 0 and class 2: accuracy 100%, sensitivity 100%, and specificity 100%
Di Lazzaro et al [55]	Sensors: IMUs (fs=50 Hz) Placement: upper back, lower back, upper arms, forearms, hands, thighs, shanks, and feet	Movit system [56]	HC (19 male and 10 female; age: mean 69, SD 11 years) PD (26 male and 10 female, age: mean 63, SD 8.7 years; H&Y scale score: mean 1.8, SD 0.6)	MDS-UPDRS III (rest tremor, postural tremor, taped alternating hand movement, leg agility, heel-toe tapping, and pull test) and TUG^k^ test	Features: power, amplitude of tremor, amplitude, asymmetry of bradykinesia and number of steps, jerk, sway area of gait, and postural stability Algorithm and model: SVM	HC and PD: accuracy: 97%
Sushkova et al [57]	Sensors: accelerometer and EMG^l^ Placement: hands (accelerometer) and the extensor muscles of the wrist and ankle joints (EMG)	No information on platforms	HC (n=8) PD (n=11 with right-hand-tremor, 7 with left-hand-tremor; H&Y scale score: 1) ET (n=8)	EMG and accelerometer were recorded for 2 postures: one with the arms straight on the armrest and the other with the arms extended forward	Classification by a separation function with wave features	HC and PD: accuracy: 100% PD and ET: accuracy: 92%
Sigcha et al [58]	Sensors: accelerometers (fz=50 Hz) Placement: wrist	Smartwatches	PD (8 male and 10 female; age: mean 64.9, SD 7.6 years; H&Y scale score 1: n=4 and H&Y scale score 2: n=10; 14 patients with tremor)	6 items from UPDRS^m^: arm rest, arm extension, finger-to-nose test, hand movements, leg agility, and postural tremor assessment	Features: time and frequency Algorithm or model: multitask CNN^n^ as a classifier with FFT^o^ data representation	Distinguish tremor (comparison with those obtained in the clinical assessment): sensitivity: 86.1%, specificity: 86.1%, and AUC^p^: 0.936
Castelli Gattinara Di Zubiena et al [59]	Sensors: IMUs Placement: head, chest, and pelvis	Wearable sensor: MTw (Xsens Technologies); platform: RotoBit^1D^ (used during a watching task)	HC (n=15; age: mean 65.2, SD 3.4 years) PD (n=19 male and 1 female; age: mean 67.7, SD 8.1 years; H&Y scale score: 1-2)	Participants stand on a robotic platform (RotoBit^1D^) in an upright position, with arms hanging vertically and feet externally rotated a preferred amount	Features: the range of motion of the head, chest, and pelvis in medio-lateral and antero-posterior directions and the reciprocal body segment rotations Algorithm or model: fine-kNN^q^	HC and PD: accuracy: 95.6% and AUC: 0.95
Lin et al [60]	IMUs (fz=100Hz) Placement: waist, chest, hands, thighs, shanks, and feet	MATRIX- wearable motion and gait quantification assessment system	PD (n=84; age: mean 58.13, SD 10.43 years; H&Y scale score: 1-1.5) ET^r^ (n=80; age: mean, 58.7, SD 13.9 years)	TUG test	Features: 33 gait and postural transition parameters, including Arm Symbolic Symmetry Index, minimum trunk angle during stand-to-sit, lean angle, maximum sagittal trunk angular velocity during sit-to-stand, and maximum angular velocity during a 180° turn Algorithm and model: SVM	PD and ET: accuracy: 84.0%, sensitivity: 85.9%, specificity: 82.1%, and AUC: 0.912
Meng et al [61]	IMUs (fz=100 Hz) Placement: head, upper thoracic, pelvis, upper arms, thighs, and shanks	Wearable full-body motion analysis system (Research Pro IMU)	HC (n=19; age range: 50-70 years) PD (n=21; age range: 50-70; H&Y scale score: 1-2)	Five cycles of 5-meter straight walking and 180° turns at a comfortable walking speed	Features derived from spatiotemporal metrics, joint kinematics, variability, asymmetry, and stability Algorithm and model: SVM with linear kernel	HC and PD: AUC: >0.65
Mirelman et al [62]	Accelerometer (fz=100 Hz) Placement: lower back	Axivity AX3 or AX6	HC (n=68; 39 male and 25 female; age: mean 58.57, SD 8.63 years) Recently diagnosed PD (n=64; 39 male an 25 female; age: mean 67.99, SD 8.46 years; H&Y scale score: 1-2) Subgroup of PD (n=33; 21 male and 12 female; age: mean 62.72, SD 8.66 years; H&Y scale score 1-2)	Real-world continuous (24-hour) digital mobility data collection for 7 days; participants in the subgroup of PD were assessed at 12-month intervals over 4 years	Algorithm and model: binary logistic regression on 14 features	HC and PD: accuracy: 81.1% and AUC: 0.87 Larger effect sizes than MDS-UPDRS for change over time (Cohen d: 0.19-0.66)
Schalkamp et al [63]	Accelerometer (fz=100 Hz) Placement: wrist	Axivity AX3	General population (n=33,009) PD (n=153) and pre-PD (n=113) in General population	Continuous monitoring over 7 days	Features: accelerometry data (daily and hourly averages), physical activity categories (sleep, sedentary, light, and MVPA^s^) Algorithm and model: Lasso logistic regression	Distinguishing PD (AUPRC^t^: mean 0.14, SD 0.04) and pre-PD up to 7 years prediagnosis (AUPRC: mean 0.07, SD 0.03) Compared with other model test (genetics AUPRC: mean 0.01, SD 0.00; *P*=.002; lifestyle AUPRC: mean 0.03, SD 0.04; *P*=.003; blood biochemistry AUPRC: mean 0.01, SD 0.00; *P*=.004; prodromal signs AUPRC: mean 0.01, SD 0.00; *P*=.004)
Shcherbak et al [64]	IMUs (fz=100 Hz) Placement: hands	The SensorTile platform	PD1 (n=54; H&Y scale score: 1-2) PD2 (n=11; H&Y: 3-4) HC (n=31)	Eleven tasks were recommended by the neurologists or taken from relevant research [65]: (1) standing up, walking, and sitting down; (2) pronation and supination of the forearm; (3) resting with arms relaxed; (4) tapping index and thumb while elbows are bent; (5) touching nose with the index finger; (6) sitting with outstretched arms; (7) tightening a nut on a bolt; (8) filling glass with water; (9) tapping fingers on a table; (10) standing with arms folded in front of the chest, with palms pointed inward; and (11) standing with arms folded in front of the chest, with palms pointed outward	Features: 33 features from statistical features, correlation features, and frequency features Algorithm and model: RF and LightGBM^u^	Best micro F1-scores of RF and LightGBM: 0.83 for HC and PD1, 0.86 for HC and PD2, and 0.78 for PD1 and PD2

^a^IMU: inertial measurement unit.

^b^fs: sampling frequency.

^c^HC: health control.

^d^H&Y: Hoehn and Yahr.

^e^iRBD: idiopathic rapid eye movement sleep behavior disorder.

^f^PD: Parkinson disease.

^g^RF: random forest.

^h^IH: idiopathic hyposmia.

^i^MDS-UPDRS III: Movement Disorder Society-Unified Parkinson’s Disease Rating Scale Part III.

^j^SVM: support vector machine.

^k^TUG: timed up and go.

^l^EMG: electromyography.

^m^UPDRS: Unified Parkinson's Disease Rating Scale.

^n^CNN: convolutional neural network.

^o^FFT: fast Fourier transform.

^p^AUC: area under the curve.

^q^kNN: k-nearest neighbor.

^r^ET: essential tremor.

^s^MVPA: moderate physical activity.

^t^AUPRC: area under precision recall curve.

^u^LightGBM: light gradient boosting machine.

Therefore, an important point identified during the paper screening process is that the rigor of the experimental design, particularly in the selection of participants, is a critical factor that must be emphasized when studying wearable sensors for the early diagnosis of PD.

We believe that studies on the early diagnosis of PD should include participants who are either in the early stage of PD, in a pre-PD phase, or healthy individuals who may develop PD upon follow-up. This is essential to ensure that study findings apply to the target population and to accurately assess the effectiveness of wearable sensors in early diagnosis of PD. Unfortunately, many of the excluded studies failed to meet this criterion, and the absence of staging information or the conflation of patients at different stages of the disease significantly undermined the credibility of their findings.

Therefore, we emphasize the importance of rigorous study design in future research on wearable sensors for the early diagnosis of PD. It is imperative to ensure that the staging information of included patients is accurate and adheres to established clinical assessment criteria, thereby guaranteeing the reliability and scientific value of the study results.

### Study Characteristics

In terms of the timeline of the selected studies, the earliest study included was published in 2018, although the search for studies began in 2013. Despite references to 3 studies on wearable sensors for the early diagnosis of PD between 2013 and 2017 in the review [37], these studies did not meet the criteria for this review and were excluded. Of the 12 included studies, 9 (75%) were published within the last 3 years, indicating a growing interest among researchers in wearable sensors for the early diagnosis of PD.

Among these studies, only 2 were longitudinal [63] or mixed methods [62] studies, while the remaining 10 were cross-sectional studies. Although cross-sectional studies are rapid, economical, and capable of handling large samples and diverse variables, they are prone to bias and error because they rely on data from a single point in time [66,67].

Regarding the purpose of the studies, 33% (4/12) of the studies focused on identification diagnosis, examining conditions such as essential tremor [57,60], idiopathic rapid eye movement sleep behavior disorder [53], and idiopathic hyposmia [54]. In addition, 2 studies [36,64] addressed the staged diagnosis of PD. One study [58] focused on identifying a specific symptom in the early stages of PD: tremor. These studies show that the use of wearable sensors in the early diagnosis of PD is evolving. Research is becoming more refined and broader in scope, progressively encompassing multiple symptoms of early PD.

Of the 12 studies, 5 (42%) [55,59,61-63] assessed the general feasibility and diagnostic performance of wearable sensors in early PD detection without targeting specific classification purposes. These studies primarily explored motion capture, gait analysis, or long-term real-world monitoring to evaluate the potential utility of wearable technologies for screening or supporting early-stage PD identification in broader contexts.

### Quality of Studies

According to the QUADAS-2 criteria, each domain (patient selection, index test, reference standard, and flow and timing) was categorized into 3 levels of risk: low, high, and unclear. The results of the quality assessment of the 12 studies included in this review are shown in Table 2.

**Table 2:** Quality assessment results of the included studies using the Quality Assessment of Diagnostic Accuracy Studies-2. The + symbol indicates low risk; the –symbol indicates high risk; the ? symbol indicates unclear risk.

Study	Risk of bias					Applicability concerns		
	Patient selection	Index test	Reference standard	Flow and timing	Patient selection		Index test	Reference standard
Arora et al [53]	+	+	+	+	+		+	+
Rovini et al [54]	+	+	+	?	+		+	+
Borzí et al [36]	–	+	+	?	+		+	+
Di Lazzaro et al [55]	+	+	+	+	+		+	+
Sushkova et al [57]	–	?	+	?	?		?	+
Sigcha et al [58]	+	+	+	+	+		+	+
Castelli Gattinara Di Zubiena et al [59]	+	+	+	+	+		+	+
Lin et al [60]	+	+	+	?	+		+	+
Meng et al [61]	+	+	+	?	+		+	+
Mirelman et al [62]	+	–	+	+	+		–	+
Schalkamp et al [63]	+	+	+	+	+		+	+
Shcherbak et al [64]	–	+	+	+	?		+	+

In terms of patient selection, out of 12 studies, 9 (75%) were rated as low risk of bias, whereas 3 (25%) [36,57,64] were rated as high risk of bias due to missing information about the age of the recipients or significant differences in the age of the participants between subgroups.

In the assessment of index tests and reference standards, most studies (11/12, 92%) were rated as low risk. The researchers used cross-validation with MDS-PD criteria or the neurologist’s clinical diagnosis as reference standard. In 1 study [62], the index test was assessed as high risk due to the use of a mixed sensor setup involving both Axivity AX3 and AX6 devices. The AX6 offers a higher sampling rate, greater storage capacity, and additional sensors compared to the AX3. In addition, the study did not specify which platform was used for each participant, limiting interpretability.

In terms of process and timing, 5 studies lacked information on the time interval between wearable sensor testing and the patient’s clinical diagnosis. These studies were rated as having an unclear risk because the early stages of PD usually last for a longer period.

Regarding applicability, except for 2 studies [57,64] that were assessed as having unclear risk due to insufficient information on patient selection and 1 study [62] assessed as high risk due to the mixing of platforms, most studies demonstrated good applicability across patient selection, index test, and reference standard and were rated as low risk.

### Wearable Sensors

A variety of wearable sensors were involved in the selected studies, including IMUs, accelerometers, gyroscopes, microphones, touch screen sensors, timers, and electromyography (EMG). The wearable sensors in the most selected studies were used to measure human motion data, except for 2 studies. One study that used EMG was conducted by Sushkova et al [57], who combined EMG with accelerometers to analyze the state of specific muscles in different postures. In another study [53], microphones, touchscreen sensors, and time sensors were used alongside IMUs as wearable sensors to measure various data, including motion and voice of the participant while using the smartphone.

Among these wearable sensors, IMUs were chosen in 8 studies. IMUs are a type of sensor that combines an accelerometer, gyroscope, and magnetometer [30,68]. The accelerometer measures linear acceleration and gravity; the gyroscope measures the rate of rotation to provide angular velocity information; and the magnetometer measures the Earth’s magnetic field to provide heading information, determining orientation relative to the Earth’s magnetic north pole [30,68,69]. The IMUs in these studies measured acceleration, angular velocity, and orientation at specific locations on the human body, with sampling frequencies ranging from 20 Hz to 00 Hz, to obtain characteristics of the participants’ gait, balance, and tremor.

The remaining 4 studies involved the use of accelerometers to measure participants’ motion data to monitor individual movement characteristics in specific postures or to detect specific symptoms in patients with early PD. Accelerometer was also used for continuous monitoring in real-world settings over long periods. Sigcha et al [58] used the accelerometer in the smartwatch to collect motion data from participants during specific movements and to detect tremor in the early stage of PD. Schalkamp et al [63] used the accelerometer for 7 days of continuous monitoring to identify the early stage of PD or pre-PD.

**Figure 2:**
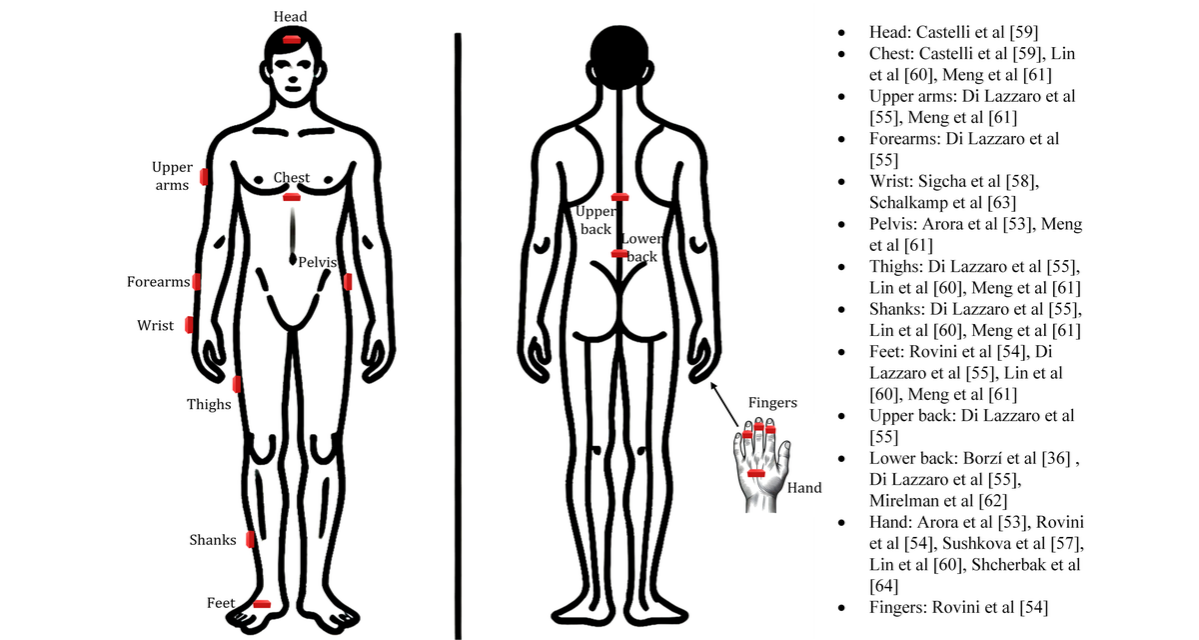
The location of wearable sensors in the selected studies.

The location of wearable sensors is critical for obtaining accurate data related to the desired measurement [70,71]. In the 12 studies included in this review, sensors were placed either in a single location or multiple body parts. For instance, in some studies, sensors were placed in a single location, such as the wrist [63] or the lower back. Other studies, such as those by Di Lazzaro et al [55] and Meng et al [61], placed sensors on multiple body parts, including the upper back, lower back, upper arms, forearms, thigh, calf, and foot of the participants. Across all the selected studies, the hands were the most common locations for sensor placement (Figure 2).

### Platforms

The relationship between wearable sensors and their corresponding platforms is mutually reinforcing. The sensors continuously collect raw data, while the platforms process, analyze, and present this information, optimizing its use in applications such as health monitoring, fitness tracking, and beyond [39,72].

In this review, various platforms were used to integrate wearable sensors into research. These platforms include systems specifically designed for scientific research and clinical applications, including the Movit system [55], MTw (Xsens Technologies) [59], and Research Pro IMU system [61]. These systems monitor the movement of multiple body parts simultaneously by integrating multiple accelerometers and gyroscopes or IMUs, enabling comprehensive analysis of whole-body movement. In addition, there are accelerometer platforms, such as Axivity AX3 and AX6 [62,63], that are developed for monitoring physical activity, sleep patterns, and other movement-related data. These platforms are suitable for continuous monitoring and were selected for use in only 2 longitudinal and mixed methods studies [62,63].

One study used self-designed platforms specifically developed for PD diagnosis and monitoring: the SensHand V1 and SensFoot V2 [54]. These specialized platforms use 100 Hz IMUs to measure hand and foot movements, respectively, and were first designed and used in PD research in 2013 [73]. Moreover, commercial platforms equipped with wearable sensors, such as smartphones and smart bracelets, were selected as test platforms due to their popularity and ease of use [53,74]. For example, Arora et al [53] acquired data on participants’ movements, voice, and reaction time through various wearable sensors (eg, IMU, microphone, and timer) integrated into the smartphone.

### Assessment Tasks

Assessment tasks are critical in clinical diagnosis to determine the patient’s status, guide treatment planning, and monitor disease progression [75,76]. For the diagnosis of PD, clinical practice relies on a variety of assessment tasks to ensure a comprehensive evaluation of the patient’s condition [77]. These tasks include both traditional clinical examinations and the application of advanced technological methods [63,77,78].

Among the studies included in this review, 6 directly used existing clinical assessment tasks, including Unified Parkinson’s Disease Rating Scale (UPDRS), MDS-UPDRS, and the timed up and go (TUG) test, for wearable sensor assessments. The UPDRS is a comprehensive tool for monitoring the burden and extent of PD [79]. Sigcha et al [58] selected 6 items of UPDRS in conjunction with accelerometers to assess and quantify tremor in early-stage PD. The MDS-UPDRS, an updated version of the UPDRS, is a comprehensive tool developed by the Movement Disorder Society [79]. It consists of 4 components, 3 of which focus on motor examination, evaluating various aspects of motor function through 18 subitems [79]. Rovini et al [54] and Di Lazzaro et al [55] selected 10 and 7 tasks (Table 1), respectively, from the MDS-UPDRS III in their studies to evaluate motor symptoms: tremor, gait, posture, and bradykinesia. The self-developed platform used by Rovini et al [54] (SensHand V1) is capable of measuring participants’ finger movements, allowing them to evaluate a wider range of items.

The TUG test, selected by Lin et al [60] and Shcherbak et al [64] as part of their assessment task, is a clinically validated method used to assess mobility and dysfunction in patients with PD [80,81]. Its duration was significantly correlated with MDS-UPDRS III scores [82].

Other studies, while not directly using clinical assessment tasks, used methods similar to or variations of the MDS-UPDRS III tasks. For example, the “Quiet stance for 30 seconds” assessment task used by Borzí et al [36] is akin to item 13 of the MDS-UPDRS III: “Posture.”

### Data Analysis

This review categories the data analysis methods used in the 12 selected studies into 3 key areas: feature extraction, algorithms or models, and performance metrics.

Feature extraction involves deriving meaningful attributes from raw sensor data to quantify various aspects of motor and nonmotor ability. Most research has focused on extracting features that reflect motor abilities, including temporal and spatial characteristics of tasks, such as gait, balance, and finger tapping, along with corresponding time and frequency features. For example, Arora et al [53] extracted features related to finger tapping, gait, and balance. Some studies have focused on specific motor features in patients with PD through clinical experiments. For instance, Di Lazzaro et al [55] examined features such as tremor power amplitude, bradykinesia amplitude asymmetry, step count, twitching, and sway area in gait and posture using the MDS-UPDRS III and TUG test. In addition, continuous real-world mobility data have been used to assess motor function. For example, Schalkamp et al [63] monitored participants continuously over 24 hours a day for 7 days via accelerometers to obtain daily and hourly averages of physical activity levels, which were then used to identify individuals with PD and those in the pre-PD stage.

While most studies have focused on motor function features, some have also addressed nonmotor function features, such as voice, response, and sleep. For instance, Arora et al [53] extracted features of the voice and reaction time of participants through a microphone and timer on a smartphone, respectively. Schalkamp et al [63] analyzed physical activity data to extract sleep features from participants.

The algorithms and models used in all 12 studies included in this review primarily consist of machine learning techniques (Table 3). As shown in Tables 2 and 3, most studies (11/12, 92%) aimed to distinguish PD or pre-PD from healthy individuals or those with related conditions. The remaining study (1/12, 8%) aimed to detect and identify the presence of the specific symptom: tremor from motion data [58]. All of these are classification problems. Consequently, most of the algorithms and models used are those commonly used for classification. SVM appeared in 4 studies, while random forest (RF) was used in 3 studies; they were the two most frequently used algorithms. SVM can handle high-dimensional data and is robust [83,84], while RF efficiently reduces the risk of overfitting and is suitable for multi-feature classification tasks containing noise, as applied by Rovini et al [54] and Shcherbak et al [64].

**Table 3:** Artificial intelligence models used in each included study and performance.

Study	Algorithms tested	Best-performing model	Performance (best)	All models and performance
Arora et al [53]	RF^a^	RF	HC^b^ versus PD^c^: sens^d^=84.6% and spec^e^=88.3%, iRBD^f^ versus PD: sens=87.5% and spec=90.1%, and HC versus iRBD: sens=91.9% and spec=90.0%	RF only
Rovini et al [54]	SVM^g^ (polynomial kernel) and RF	RF	Acc^h^=1.000 (HC versus PD) and *F1*-score=0.920 (HC versus IH^i^ versus PD)	SVM: Acc=0.967and *F1*-score=0.778 and RF: Acc=1.000and*F1*-score=0.920
Borzí et al [36]	SVM	SVM	Acc=100% (n=16, possible overfitting)	Only SVM tested
Di Lazzaro et al [55]	SVM	SVM	Acc=97%	Only SVM tested
Sushkova et al [57]	SVM and RF	RF	Acc=94%	SVM: Acc=89% and RF: Acc=94%
Sigcha et al [58]	CNN^j^ (multitask)	CNN	Acc=97% and AUC^k^=0.96	Only CNN tested
Castelli Gattinara Di Zubiena et al [59]	SVM, ANN^l^, kNN^m^, and DT^n^	SVM	Acc=95%	SVM: Acc=95%, kNN: Acc=86%, DT: Acc=83%, and ANN: Acc=84%
Lin et al [60]	SVM	SVM	Acc=91%	Only SVM tested
Meng et al [61]	SVM	SVM	AUC>0.65	Only SVM tested
Mirelman et al [62]	LR^o^	LR	Acc=81.1% and AUC=0.87 (HC versus PD)	Only LR tested
Schalkamp et al [63]	LR	LR	PD: AUPRC^p^=0.14 +0.04 or –0.04, pre-PD: AUPRC=0.07 +0.03 or –0.03	Only LR tested
Shcherbak et al [64]	LightGBM^q^ and RT	LightGBM	*F*_1_-score=0.83 (HC versus PD1), *F*_1_-score=0.86 (HC versus PD2), *F*_1_-score=0.78 (PD1 versus PD2)	Only LightGBM results reported; RF not presented

^a^RF: random forest.

^b^HC: health control.

^c^PD: Parkinson disease.

^d^sens: sensitivity.

^e^spec: specificity.

^f^iRBD: idiopathic rapid eye movement sleep behavior disorder.

^g^SVM: support vector machine.

^h^acc: accuracy.

^i^IH: idiopathic hyposmia.

^j^CNN: convolutional neural network.

^k^AUC: area under the curve.

^lj^ANN: artificial neural network.

^m^kNN: k-nearest neighbor.

^n^DT: decision tree.

^o^LR: logistic regression.

^p^AUPRC: area under precision recall curve.

^q^LightGBM: light gradient boosting machine.

Other methods include lasso logistic regression for continuous monitoring data, as used by Schalkamp et al [63], and fine k-nearest neighbor for specific feature spaces, as applied by Castelli Gattinara Di Zubiena et al [59] due to its simplicity and efficiency. In addition, Shcherbak et al [64] used light gradient boosting machine, noted for its speed and performance on large datasets. Sigcha et al [58], by contrast, used multitask convolutional neural network (CNN) to classify and identify tremor in early PD from complex motion data captured by accelerometers.

Performance metrics are critical for evaluating the validity of the algorithms and models. Common metrics used in the selected studies include accuracy, sensitivity, specificity, and area under the curve (AUC). Studies generally report high accuracy and specificity, indicating the reliability of these models. Rovini et al [54] reported 100% accuracy and specificity in distinguishing between healthy individuals and those with individuals PD, while Di Lazzaro et al [55] reported 97% accuracy for RF. Schalkamp et al [63] used area under precision recall curve to evaluate the performance of the model in their longitudinal study. Compared to AUC, area under precision recall curve focuses more on the precision (positive predictive value) and sensitivity of the positive class [63,85], making it more suitable for dealing with unbalanced datasets. The results show that machine learning models trained using accelerometer data outperform some other testing modalities (eg, genetics, lifestyle, blood biochemistry, and prodromal signs) in diagnosing patients with PD or pre-PD.

## Discussion

### RQ 1: What Types of Wearable Sensors Are Being Used for the Early Diagnosis of PD?

In the 12 selected studies, researchers primarily used IMUs and accelerometers as wearable sensors for the early diagnosis of PD. In addition, a small number of studies used EMG as well as wearable sensors such as timer and microphone integrated into smartphones.

IMUs were the most frequently chosen wearable platform, featuring in 67% (8/12) of the studies. In 6 (75%) of these 8 studies, motor characteristics were measured during specific actions. Researchers placed the IMUs on various parts of the body, including the wrist, waist, feet, and hands, to obtain motion data under specific actions to reflect whether the participants exhibited the motor symptoms of patients with early-stage PD, including gait, balance, and tremor. A common feature of these studies is the use of clinical assessments in combination with dedicated wearable platforms and multiple IMUs used simultaneously. This approach, although prescriptive in terms of movement form and demanding in terms of testing, allows for the testing of all types of typical motor symptoms associated with PD.

Accelerometer is the other main sensor of choice, and the studies involved include both clinical testing modalities and long-term monitoring. Accelerometer is characterized by the small number of sensors used, with 3 studies [58,62,63] involving a single location, making them suitable for real-time monitoring in the real world. For example, Schalkamp et al [63] used wrist-worn accelerometers for 7 days of continuous monitoring.

Overall, it can be noted that most wearable sensors in the selected studies were motion data sensors. However, there are also a variety of sensors that may have applications in early diagnostic studies of PD that were not identified in the included studies, especially for monitoring nonmotor symptoms in early PD. For example, pressure sensors, electroencephalography (EEG), and photoplethysmography (PPG) volumetric tracing sensors. Wearable pressure sensors, for instance, may be used to monitor not only motor symptoms, such as gait and tremor in early-stage PD [86], but also nonmotor symptoms. Gastrointestinal problems (eg, constipation) are one of the common nonmotor symptoms in early-stage PD [87]. Tai et al [88] designed a wearable pressure sensor using conductive hydrogel spheres, which could theoretically be used to monitor autonomic function in the gastrointestinal tract.

The use of EEG in the early diagnosis of PD has been widely demonstrated [89-91]. EEG was included in the initial search strategy due to the theoretical applicability of wearable EEG devices in real-world, mobile health monitoring scenarios. However, the EEG platforms used in the studies included in this review were clinical-grade systems, that is, multielectrode, multichannel amplifiers with lead caps [89-91]. These types of EEG platforms severely restrict the participant’s physical activity when in use and, therefore, do not fall into the category of wearable sensors. In recent years, wearable EEG platforms have matured in terms of design, development, and practical application [92,93], and thus, wearable EEG can theoretically be applied to the early diagnosis of PD. However, existing wearable EEGs has significantly fewer electrodes compared to clinical EEG platforms. As a result, they are inferior to clinical EEG platforms in terms of spatial resolution of signal acquisition, signal quality, and stability. These differences may present limitations in application in early diagnosis of PD. The PPG sensor is a wearable sensor that measures changes in blood volume in tissue microvascular beds [94]. Applications in electrocardiography, heart rate, and blood pressure monitoring have attracted attention and have been used in smart platforms such as smartwatches and smartphones to detect physiological indicators and for disease diagnosis [94-96]. For example, Riaz et al [97] used PPG in combination with neural networks to noninvasively estimate blood pressure in participants, and Lan et al [98] analyzed heart rate variability signals acquired by PPG in both time and frequency domains to predict hypertension. Although no studies have applied PPG sensors specifically for the early diagnosis of PD, some have explored the relationship between heart rate variability, blood pressure, and early diagnosis of PD [99,100]. Therefore, PPG sensors have the potential to be applied to the early diagnosis of PD.

### RQ 2: What Methods and Technologies Are Used in These Studies, Including Wearing Styles, Types of Data Acquired, and Methods of Data Analysis and Processing?

From the results, it can be observed that the wearable sensors involved in the studies of early diagnosis of PD used a variety of wearing styles, data types, and data analysis.

#### Wearing Styles

The placement and design of wearable sensors are critical for accurate data collection. The sensors used in the studies were worn in various ways, either distributed across the body (eg, the wrist, waist, feet, head, and hands) or in a single location. The wearing styles used in different studies correlate with their target data type and the early symptoms of PD.

For example, studies by Rovini et al [54] and Sushkova et al [57] have shown that placing IMUs on the hands or fingers can measure fine motor movements to monitor bradykinesia and tremor, which are symptoms of PD. Conversely, Borzí et al [36] and Lin et al [60] placed IMUs on the feet and waist to assess gait and balance. Comprehensive body assessment requires placing sensors on multiple parts of the body, as seen in the studies by Di Lazzaro et al [55] and Meng et al [61], who placed sensors on the upper back, lower back, upper arms, forearms, thighs, calves, and feet to detect multiple motor symptoms of PD. For conditions requiring continuous assessing, sensors were placed at a single location to capture daily activities without disturbing the participants; for example, Schalkamp et al [63] used wrist-worn sensors, while Mirelman et al [62] placed sensors on the lower back.

#### Types of Data Acquired

The wearable sensors used in the studies collected a variety of data types, including motion data, such as acceleration and angular velocity, as well as physiological parameters, such as EMG [57], voice, and reaction time [53]. Motion data, acquired by IMUs and accelerometers, reflect various motor symptoms in PD, such as gait, balance, tremor, and bradykinesia. EMG sensors record muscle electrical signals to understand muscle activity and tremor. Timers, microphones, and touch screen sensors in smartphones are used to acquire speech and reaction time data to detect nonmotor symptoms of PD, such as dysarthria and cognitive dysfunction.

#### Methods of Data Analysis and Processing

Feature extraction is a crucial initial step in studies involving wearable sensors, as it involves extracting meaningful attributes from the raw data acquired by the sensors. As shown in Table 1, the features chosen for extraction in various studies are related to symptoms commonly observed in early-stage PD. For example, when assessing motor symptoms such as gait, balance, and tremor in PD, the extracted features include features of time and frequency and specific features.

Borzí et al [36] extracted features of time and frequency to classify healthy individuals and those with early-stage PD. Lin et al [60], using IMUs combined with the TUG test, extracted specific features—such as arm symbolic symmetry index, stand-to-sit trunk minimum, lean angle, sit-to-stand trunk maximum sagittal angular velocity, and 180° turn maximum sagittal angular velocity—to distinguish early-stage PD from essential tremor. However, the features selected in different studies vary somewhat and lack uniformity, indicating that the consistency and standardization of feature extraction need to be addressed.

Machine learning is widely used in research to improve classification accuracy. According to research findings, SVM and RF are the most used and best-performing algorithms in existing studies. It is important to note that multiple algorithms are usually tested in research, with the optimal results being selected. For example, Di Zubiena et al [59] attempted to classify individuals with early-stage PD and healthy individuals using k-nearest neighbor, decision tree, SVM, and artificial neural network. The results indicated that SVM performed the best. Conversely, logistic regression models were chosen for classification by 2 studies involving continuous monitoring data due to their speed and performance on large datasets.

In addition, deep learning models such as CNN have been used for the representation and classification of complex data. Sigcha et al [58] used multitasking CNN to classify the tremor in early-stage PD from accelerometer data, illustrating the potential of deep learning in early diagnosis.

### RQ 3: What Are the Accuracy, Reliability, Utility, and Limitations of Wearable Sensors in the Early Diagnosis of PD?

The 12 selected studies used various wearable sensors and methods to evaluate the accuracy, reliability, and utility of wearable sensors in the early diagnosis of PD for different purposes.

#### Accuracy and Reliability

The results of these studies indicate that wearable sensors achieve high accuracy and reliability in the early diagnosis of PD, including screening, differential diagnosis, and graded diagnosis of early-stage PD or pre-PD. For example, in screening, Schalkamp et al [63] found that wearable sensors (accelerometer) outperformed genetics, lifestyle, blood biochemistry, and prodromal signs in terms of diagnostic accuracy for early-stage PD or pre-PD through a long-term (7 years) follow-up study. Regarding differential diagnosis, Arora et al [53] used various types of wearable sensors (IMUs, timers, microphones, and touchscreen sensors) integrated into smartphones and analyzed using RF. They achieved a sensitivity of 87.5% and a specificity of 90.1% in distinguishing early-stage PD from iRBD.

However, there were variations in accuracy between the studies, which may be attributed to the use of different wearable sensors, assessments, and data analysis methods. In addition, the reliability results reported in the studies may have been affected because 83% (10/12) of the studies were cross-sectional and did not include the undiagnosed group, and 7 (58%) studies had small sample sizes.

#### Utility

Most of the studies (10/12, 83%) were experimental investigations of wearable sensors for the early diagnosis of PD. The tests used in these studies were complex, involving multiple clinical tests, multilocation sensors, large amounts of data acquisition, and intricate data analysis. For example, in the study by Di Lazzaro et al [55], participants were required to complete 7 tests, each involving different IMU placements and quantities. During the TUG test, up to 9 IMUs were placed at different locations on the body. These complex tests may affect the portability and simplicity of wearable sensors in clinical settings, limiting their usefulness.

However, 2 studies used wearable sensors placed at a single body location and conducted testing in nonclinical settings. For example, Mirelman et al [62] used an accelerometer to continuously monitor participants in real-world settings for 24 hours a day over 7 days, achieving high screening accuracy (health control/PD: AUC=0.87). This study fully used the portability, simplicity, and continuous monitoring capabilities of wearable sensors and showed the potential of wearable sensors for real-world applications in the early diagnosis of PD.

### Limitations and Prospect

On the basis of the 12 selected studies, it is evident that wearable sensors have significant potential for the early diagnosis of PD, though there are some limitations. First, as the research on wearable sensors for early PD diagnosis is still in the exploratory experimental phase, most of the studies (10/12, 83%) used a cross-sectional design, meaning data were collected at a single time point. Cross-sectional design limits the ability to assess the effectiveness and stability of wearable sensors for early PD diagnosis. Furthermore, the lack of longitudinal or mixed methods studies highlights a significant gap in understanding the long-term effectiveness and stability of wearable sensors in the early diagnosis of PD. In addition, these cross-sectional studies did not include the creation of unknown diagnostic groups, meaning the generalizability of the models to untested datasets was not validated, which could lead to a decline in the predictive performance of the models in real-world applications, especially when accuracy and reliability are difficult to ensure with undiagnosed populations [101]. Although cross-validation was used in all cross-sectional studies, it has been shown that cross-validation cannot fully replace the need to introduce unknown diagnostic groups [102].

Second, the sample size of patients with early-stage PD in the 7 studies was <30, which constitutes a small sample size. Small sample sizes can lead to statistical issues, reduce the ability to detect meaningful differences, and increase the risk of type I and type II errors [103]. Moreover, small sample sizes suffer from data underrepresentation, overfitting, and unpredictable errors in machine learning models [104]. For example, Borzí et al [36] achieved 100% accuracy in classifying patients with early-stage PD and healthy individuals with their developed model, but they only had 16 patients with early-stage PD, which does not exclude the risk of overfitting.

Furthermore, early-stage PD exhibits motor and nonmotor symptoms, such as loss or diminution of smell, sleep disturbances, constipation, and tremor [17,20].

Among the 12 included studies, 9 (75%) focused on motor symptoms, such as gait, tremor, postural instability, and bradykinesia. The remaining 3 studies (25%) [53,63,64] examined both motor and nonmotor symptoms, including voice, reaction time, sleep indicators, and physical activity. This distribution indicates that the existing literature predominantly emphasizes motor symptoms of PD, whereas nonmotor symptoms remain relatively underexplored. This may be due to the selected studies predominantly using sensors that acquire motor data. Future studies should consider using wearable sensors capable of detecting nonmotor symptoms, such as wearable EEG and PPG sensors.

Current studies have not adequately demonstrated the application of wearable sensors for the early diagnosis of PD. The methods used in existing studies, including wearing styles, assessment, and data analysis techniques, have limitations based on their research content and are challenging to expand to clinical diagnostic applications [105]. There are differences among studies in the selection of testing methods, sensor placement, and data collection and analysis, which makes it difficult to compare experimental results and promote their use across a broad population, except for some impact on individual case diagnosis.

In addition, the portability of wearable sensors is an advantage in the early diagnosis of PD, enabling real-time monitoring in real-world settings. However, the 12 selected studies had a low likelihood of real-world application due to various issues. In 10 (83%) of these 12 studies, limitations in sensor placement, assessments, and platforms used restrict their use in laboratories or hospitals. For example, Meng et al [61] and Di Lazzaro et al [55] used whole-body multisite wearables. Sigcha et al [58] used a single location and a commercially available smart platform; their testing method used the clinical testing program UPDRS, which requires professional testers (medical personnel), limiting its practicality for real-world application. The remaining 2 studies [62,63] used a single sensor placement and a real-time testing approach, but the testing platforms were specialized research testing platforms, reducing their potential for practical application.

Finally, among the selected studies, AI, including machine learning and deep learning algorithms, has been increasingly adopted to enhance the diagnosis of early PD using wearable sensors. While these methods have demonstrated advantages in handling high-dimensional time-series data collected from complex wearable devices, their current applications remain largely exploratory. The algorithms used in the reviewed studies vary significantly in terms of algorithm selection, feature extraction, and evaluation approaches, which limits comparability and generalizability. Furthermore, most studies are based on relatively small sample sizes, increasing the risk of overfitting and reducing model robustness. In addition, few AI-based models have been validated in real-world or longitudinal settings.

To complement the studies included in this review, we further examined recent publications in 2024 that explored the use of wearable sensors for diagnosing the early stage of PD. Among these, 2 studies met the inclusion criteria in our systematic review.

The first study, by Adams et al [106], recruited 82 patients with early-stage PD (H&Y scale score ≤2; disease duration <2 years) and 50 healthy controls. Participants were followed longitudinally for 12 months using commercial smartwatches and smartphones. RF classification was developed based on data, achieving high diagnostic performance with an accuracy of 92.3%, sensitivity of 90.0%, and specificity of 100%. This study contributes significantly to the limited number of longitudinal studies.

The second study, by Choi et al [107], used the Xsens (IMUs) to collect data during the 6-minute walk test from 78 patients with PD (H&Y scale score ≤2). CNN was used to model signals from multiple body locations, with gyroscope data from the lumbar region achieving the highest classification accuracy of 83.5%.

In contrast, several other recent studies exhibited significant limitations in participant selection [108-110]. These studies either failed to restrict participants to early-stage PD or did not provide sufficient clinical staging and medication information. Although methodologically innovative and indicative of the promise of wearable sensors and AI for diagnosing PD, the lack of strict control over disease staging compromises the clinical interpretability and comparability of their findings. These observations highlight the critical importance of accurate participant staging and sample homogeneity in wearable sensor research on the early diagnosis of PD.

### Future Directions

Future research may benefit from using more longitudinal or mixed methods designs to investigate the long-term utility and diagnostic stability of wearable sensors in the early detection of PD. Broadening the scope to include nonmotor symptoms could contribute to a more comprehensive diagnostic framework. In addition, integrating diverse sensing modalities and encouraging standardization in sensor placement and data processing may enhance comparability across studies. When exploring real-world applications, researchers are encouraged to design sensor placement and assessment tasks that remain simple and practical enough for use in everyday environments, avoiding unnecessary complexity that could hinder usability.

### Conclusions

This study summarizes the research on the use of wearable sensors for the early diagnosis of PD over the past decade. The findings highlight the considerable potential of wearable sensors in this area. Nevertheless, the existing studies are limited by cross-sectional designs and small sample sizes, which may affect the generalizability and clinical applicability of their results. Future research should prioritize longitudinal and clinically validated approaches to further establish the diagnostic value of wearable sensors in early-stage PD.

## Appendix

Multimedia Appendix 1 PRISMA (Preferred Reporting Items for Systematic Reviews and Meta-Analyses) checklist.

Multimedia Appendix 2 The search strategies for 4 databases (PubMed, IEEE Xplore, Scopus, and Web of Science).

## Abbreviations

AI: artificial intelligence
AUC: area under the curve
CNN: convolutional neural network
EEG: electroencephalography
EMG: electromyography
IMU: inertial measurement unit
MDS-PD: Movement Disorder Society Clinical Diagnostic Criteria for Parkinson’s Disease
MDS-UPDRS: Movement Disorder Society-Unified Parkinson’s Disease Rating Scale
PD: Parkinson disease
PPG: photoplethysmography
PRISMA: Preferred Reporting Items for Systematic Reviews and Meta-Analyses
QUADAS-2: Quality Assessment of Diagnostic Accuracy Studies-2
RF: random forest
RQ: research question
SVM: support vector machine
TUG: timed up and go
UPDRS: Unified Parkinson’s Disease Rating Scale

## Data Availability

Data sharing is not applicable to this paper as no datasets were generated or analyzed during this study.
