# Supplementary material for: Evaluating the Utility of Wearable Sensors for the Early Diagnosis of Parkinson Disease: Systematic Review
Source: J Med Internet Res. 2025 Jul 21;27:e69422. doi: 10.2196/69422 (PMC12322615; doi:10.2196/69422)
Supplement: Multimedia Appendix 2 [file jmir_v27i1e69422_app2.pdf]

**Supplementary Material**

Research strategy for each database.

| Database | Search String |
|---|---|
| PubMed | (("Parkinson" OR "pre-Parkinson") AND ("wearable" OR "inertial" OR "accelerometer" OR "acceleration" OR "gyroscope" OR "EMG" OR "EEG" OR "ECG" OR "GSR" OR "pressure" OR "clothes" OR "smartphone" OR "smartwatch" OR "glove") AND ("early diagnosis" OR "early detection" OR "early stage" OR "timely diagnosis" OR "early identification" OR "pre-diagnostic" OR "prompt diagnosis" OR "initial detection" OR "anticipatory diagnosis" OR "prodromal" OR "pre-symptomatic" OR "preclinical" OR "incipient" OR "pre-manifest" OR "subclinical" OR "latent")) |
| Scopus | TITLE-ABS-KEY(("Parkinson" OR "pre-Parkinson") AND ("wearable" OR "inertial" OR "accelerometer" OR "acceleration" OR "gyroscope" OR "EMG" OR "EEG" OR "ECG" OR "GSR" OR "pressure" OR "clothes" OR "smartphone" OR "smartwatch" OR "glove") AND ("early diagnosis" OR "early detection" OR "early stage" OR "timely diagnosis" OR "early identification" OR "pre-diagnostic" OR "prompt diagnosis" OR "initial detection" OR "anticipatory diagnosis" OR "prodromal" OR "pre-symptomatic" OR "preclinical" OR "incipient" OR "pre-manifest" OR "subclinical" OR "latent")) |
| Web of Science | TS=(("Parkinson" OR "pre-Parkinson") AND ("wearable" OR "inertial" OR "accelerometer" OR "acceleration" OR "gyroscope" OR "EMG" OR "EEG" OR "ECG" OR "GSR" OR "pressure" OR "clothes" OR "smartphone" OR "smartwatch" OR "glove") AND ("early diagnosis" OR "early detection" OR "early stage" OR "timely diagnosis" OR "early identification" OR "pre-diagnostic" OR "prompt diagnosis" OR "initial detection" OR "anticipatory diagnosis" OR "prodromal" OR "pre-symptomatic" OR "preclinical" OR "incipient" OR "pre-manifest" OR "subclinical" OR "latent")) |
| IEEE Xplore | (("Parkinson" OR "pre-Parkinson") AND ("wearable" OR "inertial" OR "accelerometer" OR "acceleration" OR "gyroscope" OR "EMG" OR "EEG" OR "ECG" OR "GSR" OR "pressure" OR "clothes" OR "smartphone" OR "smartwatch" OR "glove") AND ("early diagnosis" OR "early detection" OR "early stage" OR "timely diagnosis" OR "early identification" OR "pre-diagnostic" OR "prompt diagnosis" OR "initial detection" OR "anticipatory diagnosis" OR "prodromal" OR "pre-symptomatic" OR "preclinical" OR "incipient" OR "pre-manifest" OR "subclinical" OR "latent")) |
